# OEsophageal Ion Transport Mechanisms and Significance Under Pathological Conditions

**DOI:** 10.3389/fphys.2020.00855

**Published:** 2020-07-16

**Authors:** Eszter Becskeházi, Marietta Margaréta Korsós, Bálint Erőss, Péter Hegyi, Viktória Venglovecz

**Affiliations:** ^1^Department of Pharmacology and Pharmacotherapy, University of Szeged, Szeged, Hungary; ^2^Institute for Translational Medicine, Szentágothai Research Centre, Medical School, University of Pécs, Pécs, Hungary; ^3^Division of Gastroenterology, First Department of Medicine, Medical School, University of Pécs, Pécs, Hungary; ^4^First Department of Medicine, University of Szeged, Szeged, Hungary

**Keywords:** ion transport, oesophagus, Barrett oesophagus, eosinophilic oesophagus, oesophageal cancer

## Abstract

Ion transporters play an important role in several physiological functions, such as cell volume regulation, pH homeostasis and secretion. In the oesophagus, ion transport proteins are part of the epithelial resistance, a mechanism which protects the oesophagus against reflux-induced damage. A change in the function or expression of ion transporters has significance in the development or neoplastic progression of Barrett’s oesophagus (BO). In this review, we discuss the physiological and pathophysiological roles of ion transporters in the oesophagus, highlighting transport proteins which serve as therapeutic targets or prognostic markers in eosinophilic oesophagitis, BO and esophageal cancer. We believe that this review highlights important relationships which might contribute to a better understanding of the pathomechanisms of esophageal diseases.

## Introduction

Ion and water transport play a crucial role in the development of gastrointestinal (GI) diseases, such as cystic fibrosis or diarrhea. Therefore, a number of studies have been conducted to identify the presence and function of transport proteins on GI cells, especially in exocrine glands, the stomach and the colon (Toth-Molnar et al., 2007; Venglovecz et al., 2008, 2011; Farkas et al., 2011; Hegyi et al., 2011; Czepan et al., 2012; Judak et al., 2014) both under physiological and pathophysiological conditions. In contrast, the literature on ion transport in the oesophagus is limited. The oesophageal epithelium (OE) is built up from squamous epithelial cells (SECs) arranged in stratified layers. Although the OE is not a typical secretory epithelium, its ion transport processes are of great importance with regard to epithelial resistance (Orlando, 1986; Goldstein et al., 1994; Gunther et al., 2014). OE resistance is an important defense mechanism which prevents cells from reflux-induced acidosis (Fujiwara et al., 2005). OE resistance involves a number of factors, such as ion transport processes through apical and basolateral membranes. Disturbances of oesophageal ion transport might play a role in certain pathological conditions, such as eosinophilic oesophagitis (EoO), Barrett’s oesophagus (BO) and oesophageal cancer (OC). This review summarizes our knowledge of the expression and function of oesophageal ion transporters and elucidates their role in oesophageal diseases. We believe that this review highlights important relationships which might contribute to a better understanding of the pathomechanisms of oesophageal diseases.

## Structure of the Oesophagus

**FIGURE 1:**
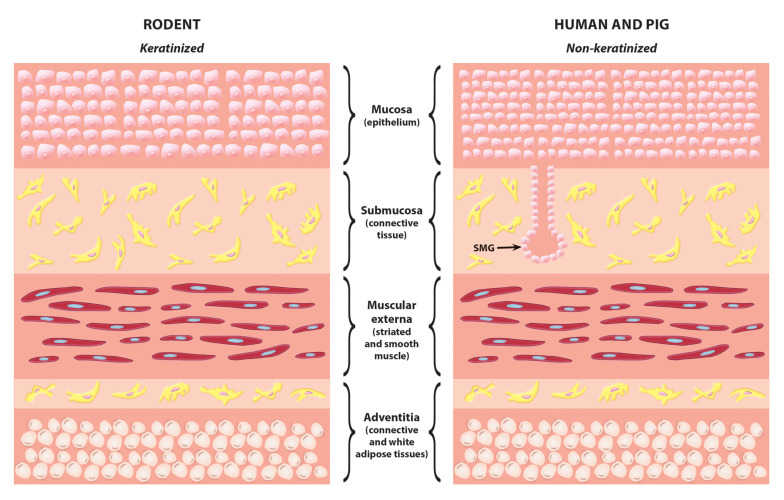
Histological comparison of rodent and human/pig oesophagus. The most common difference between rodent and human/pig oesophagus is that in rodents the mucosa layer is thinner and the lack of submucosal glands. SMG, submucosal gland.

The oesophagus consists of four layers, namely the mucosa, submucosa, muscularis externa, and adventitia. Epithelial cells are located in the mucosa and submucosa layers. The mucosa layer is composed of squamous epithelial cells arranged in several rows. There are differences in the thickness and keratinization of the mucosa between species (Figure 1). In the oesophagus of rodents, squamous cells are arranged to 4 to 5 layers and are typically keratinised. In humans and pig’s oesophagus, squamous cells are located in several layers, up to 10–15, and the cells are not keratinised. The submucosa layer contains connective tissue cells, blood and lymphatic vessels, nerves, and glands. Interestingly, there are no glands in the oesophagus of rodents, while they occur in large numbers in the oesophagus of humans and pigs. The submucosal glands (SMGs) are tubuloacinar glands that occur scattered throughout the submucosa. SMGs secrete mainly mucus, HCO_3_^–^ and growth factors, and their primary function is to lubricate the oesophagus and to protect it from the damaging effects of the refluxed acid. The submucosa layer is followed by the muscular externa that is composed of smooth and striated muscle, while adventitia is mostly composed of connective and adipose tissue. Ion mechanisms on SECs have been primarily studied in rabbit, whereas pigs proved to be a suitable animal model for studying ion transport processes associated with SMGs. The next section describes the ion transporters so far identified on SECs and SMGs.

## Presence and Role of Ion Transporters Under Physiological Conditions

### Cl^–^ Channels

Chloride (Cl^–^) transport is essentially a two-step process that involves the entry of Cl^–^ into the cell across the basolateral membrane and its secretion into the lumen through the apical membrane (Frizzell and Hanrahan, 2012). These processes involve various transport proteins, of which Cl^–^ is usually taken up by cells through the Na^+^/K^+^/2Cl^–^ co-transporter (NKCC) and released to the lumen through the cystic fibrosis transmembrane conductance regulator (CFTR). Epithelial Cl^–^ transport provides an electrical driving force for the paracellular transport of Na^+^ from the basolateral side to the lumen. As a result, the osmotic pressure in the lumen increases, which promotes the movement of water into the lumen (Frizzell and Hanrahan, 2012). Oesophageal Cl^–^ transport in the squamous epithelium and SMGs was mainly investigated by Abdulnour-Nakhoul et al. (2002) (Tables 1, 2). Using isolated rabbit OE, they showed the presence of Cl^–^ conductance at both the basolateral and the apical membrane of oesophageal epithelial cells (OECs) (Abdulnour-Nakhoul et al., 2002). The basolateral Cl^–^ conductance was Ca^2+^-dependent and flufenamate-sensitive. However, the CFTR activator, cyclic adenosine monophosphate (cAMP), or the anion exchanger (AE) inhibitor, 4,4′-diisothiocyano-2,2′-stilbenedisulfonic acid (DIDS), did not affect Cl^–^ conductance. In addition, Cl^–^ transport was independent of the presence of extracellular HCO_3_^–^, indicating that basolateral Cl^–^ conductance is believed to be a Ca^2+^-activated Cl^–^ channel (CaCC). In contrast, apical Cl^–^ conductance played only a minor role in Cl^–^ transport indicating that there is no significant transcellular Cl^–^ transport across squamous cells. Transcellular Cl^–^ transport has greater importance in SMGs, where fluid secretion is more significant. Apical Cl^–^ transport in SMGs can be linked to the CFTR Cl^–^ channel (Abdulnour-Nakhoul et al., 2011). Using different approaches, the CFTR has been detected in the acinar cells and at the apical membrane of intra- and interlobular ductal cells of pig oesophageal SMGs. In addition, using different activators and inhibitors it has been demonstrated that the CFTR Cl^–^ channel is functionally active and is expected to be involved in HCO_3_^–^ secretion in two ways: the CFTR (i) changes its permeability and secretes HCO_3_^–^ directly into the lumen and (ii) provides extracellular Cl^–^ for the function of the apical Cl^–^/HCO_3_^–^ exchanger, similarly, to the pancreas or the salivary gland (Lee et al., 2012). The transcellular transport of Cl^–^ is mediated by the basolateral AE2, through which Cl^–^ enters the cell.

**TABLE 1:** Expression of ion transporters in oesophageal squamous epithelial cells (SECs).

**Ion transporters in oesophageal squamous epithelial cells (SECs)**					
**Family**	**Type or isoform**	**Physiological role**	**Localization**	**Species**	**References**
Cl^–^ channel	CaCC	Regulates intracellular Cl^–^ concentration	Basolateral	Rabbit	Abdulnour-Nakhoul et al., 2002
K^+^ channel	N/A	Regulates transepithelial Na^+^ uptake and cell volume and also modulates RVD by K^+^ efflux	Basolateral	Rabbit	Goldstein et al., 1993; Khalbuss et al., 1993; Snow et al., 1993
				Human	Orlando et al., 2002
KCC	N/A	Secondary regulatory mechanism of RVD	Basolateral	Human	Orlando et al., 2002
NKCC	NKCC1	Operates at acidic pH and regulates cell volume	N/A	Rabbit	Tobey et al., 1995
NHE	NHE1	Causes cellular alkalisation by extruding H^+^ out of the cells	Basolateral	Rabbit	Powell et al., 1975; Layden et al., 1990, 1992; Abdulnour-Nakhoul et al., 1999; Shallat et al., 1995
				Rat	Shallat et al., 1995
				Human	Goldman et al., 2010; Guan et al., 2014; Laczko et al., 2016; Ariyoshi et al., 2017
AE	Na^+^-dependent	Causes cell alkalisation, mediates Na^+^, Cl^–^ and HCO_3_^–^ transport through the plasma membrane	Basolateral	Rabbit	Bonar and Casey, 2008; Tobey et al., 1993
	Na^+^-independent	Causes cell acidification, mediates Cl^–^ and HCO_3_^–^ transport through the plasma membrane			
non-selective cation channel	ENaC (?)	Equally permeable for monovalent cations (Li^+^, Na^+^ and K^+^), the exact role is not known	Apical	Rabbit	Awayda et al., 2004

*AE, anion exchanger; CaCC, Ca^2+^-activated Cl^–^ channel; KCC, KCl co-transporter; NKCC, Na^+^/K^+^/2Cl^–^ co-transporter; NHE, Na^+^/H^+^ exchanger; RVD, regulatory volume decrease; N/A, not available.*

**TABLE 2:** Expression of ion transporters in oesophageal submucosal glands (SMGs).

**Ion transporters in oesophageal submucosal glands (SMGs)**					
**Family**	**Type or isoform**	**Physiological role**	**Localization**	**Species**	**References**
Cl^–^ channel	CFTR	Contributes to luminal HCO_3_^–^ secretion	Apical	Pig	Abdulnour-Nakhoul et al., 2011
NKCC	NKCC1	Regulates cell volume and provides Cl^–^ for HCO_3_^–^ secretion	Basolateral		
AE	AE2	Facilitates the influx of Cl^–^ and the efflux of HCO_3_^–^	Basolateral		
	SLC26A6	Mediates HCO_3_^–^ transport into the lumen	Apical		
NBC	NBC1	Contributes to luminal HCO_3_^–^ transport	Basolateral		Abdulnour-Nakhoul et al., 2005, 2011

*AE, anion exchanger; CFTR, cystic fibrosis transmembrane conductance regulator; NBC, Na^+^/HCO_3_^–^ co-transporter; NKCC, Na^+^/K^+^/2Cl^–^ co-transporter.*

### K^+^ Channels

Potassium (K^+^) channels have an extensive family, which includes voltage-dependent K^+^ channels, Ca^2+^-activated K^+^ channels, or ATP-regulated K^+^ channels (Heitzmann and Warth, 2008). Epithelial K^+^ channels can be found on both the basolateral and luminal membranes of the epithelial cells where they serve different functions. Voltage-dependent K^+^ channels are usually expressed on the basolateral membrane, where they determine the membrane potential of the cell, provide an electrochemical driving force for the transport of other ions, such as Na^+^ or Cl^–^ and also play a role in the recycling of K^+^ or the regulation of cell volume (Cotton, 2000). K^+^ channels on the apical membrane mediate the secretion of K^+^ into the lumen and therefore determine the K^+^ concentration of the secreted fluid. Using chamber studies have shown the presence of K^+^ conductance at the basolateral membrane of rabbit OECs (Goldstein et al., 1993; Khalbuss et al., 1993). The K^+^ channel is active under basal conditions and regulates transepithelial Na^+^ uptake and cell volume after osmotic stress. The basolateral K^+^ channel has also been shown to be important in regulatory volume decrease (RVD) in isolated rabbit oesophageal cells and human OECs (Snow et al., 1993; Orlando et al., 2002). RVD plays role in the restitution of cell volume after an osmotic swelling. In most cases, RVD is mediated through the efflux of K^+^ and Cl^–^ through the basolateral membrane, followed by water transport. Snow et al. (1993) confirmed the significance of K^+^ efflux in RVD and also showed that acidic pH inhibits K^+^ channel activity, indicating that reduced RVD is probably involved in the reflux-induced oesophageal injury. In addition, using pharmacological inhibitors (e.g., DIDS and diphenylamine-2-carboxylate) or intracellular Cl^–^ removal, the authors showed that RVD also depends on Cl^–^ efflux (Snow et al., 1993). Orlando et al. (2002) made similar observations in human oesophageal cells, with the difference that they also identified a later, second mechanism underlying RVD regulation: using R(+)-butylindazone, a KCl co-transporter (KCC) inhibitor, they detected late RVD inhibition (Orlando et al., 2002). KCC is an electroneutral transporter that mediates the coupled transport of 1 K^+^ and 1 Cl^–^ for which the energy is provided by the K^+^ gradient maintained by the Na^+^/K^+^ ATPase pump (Haas and Forbush, 1998, 2000). The KCC family has four isoforms (KCC1-4) which show high levels of homology in their amino acid sequence. Although KCC1 ubiquitously expressed, the presence of KCC2 and -3 mainly limited to the nervous system. Abnormal regulation of KCC genes leads to the development of hematological diseases such as sickle cell anemia, which causes cellular dehydration in red blood cells (Rees et al., 2015). In the oesophagus, K^+^ and Cl^–^ transport across the basolateral membrane of SECs present a protective mechanism by which cells try to maintain the cell volume against hypo-osmotic shock under acidic pH which has a significance in reflux disease (Table 1).

### Na^+^/K^+^/2Cl^–^ Co-Transporter

The Na^+^/K^+^/2Cl^–^ co-transporter (NKCC) mediates the electroneutral uptake of 1 Na^+^, 1 K^+^ and 2 Cl^–^ ions across the plasma membrane (Russell, 2000). The NKCC has two isoforms in humans (NKCC1 and NKCC2), which are encoded by two different genes (*SLC12A1* and *SLC12A2*). The NKCC is primarily located on the basolateral membrane of various cells and plays a role in several physiological functions, such as fluid secretion in exocrine glands (Haas and Forbush, 2000) smooth muscle cell contraction (Akar et al., 2001) and early neuronal development (Ben-Ari et al., 2007). In most cells, NKCC interacts with KCC. The effect of the two transporters is opposite. KCC mediates Cl^–^ efflux, whereas NKCC promotes the uptake of Cl^–^. The coordinated operation of the two transporters ensures the proper Cl^–^ concentration in the cell. NKCC activity is greatly influenced by intracellular Cl^–^ concentration, which acts as a negative feedback mechanism (Rocha-Gonzalez et al., 2008). The mechanism by which cytoplasmic Cl^–^ regulates NKCC is not fully understood, but probably Cl^–^ is able to modify the phosphorylated state of the cotransporter. Loss of function mutations of NKCC2 leads to the development of type 1 Bartter’s syndrome, in which the reabsorption of salt is reduced in the thick ascending limb of Henle’s loop (Castrop and Schiessl, 2014). In oesophageal SECs, the NKCC is primarily involved in cell volume regulation (Tobey et al., 1995). Functional studies on rabbit OE have shown that bumetanide-sensitive Na^+^/K^+^/Cl^–^ transport is activated by acidic pH, is linked to the NKCC and induces cell oedema by ion uptake (Tobey et al., 1995). K^+^ and Cl^–^ conductances work in opposite directions and promote cell shrinkage. NKCC1 is present in the human oesophagus, in the lower and middle layers of squamous cells (Shiozaki et al., 2014a). The NKCC is also present in SMGs, where it plays an essential role in fluid secretion (Abdulnour-Nakhoul et al., 2011). Immunostaining of pig oesophagus shows strong, positive staining against NKCC1 antibody on the basolateral membrane of mucous cells and interlobular ducts. Bumetanide, a specific NKCC1 inhibitor, inhibits HCO_3_^–^ secretion, indicating that NKCC1 is involved in the process, presumably by providing the Cl^–^ necessary for HCO_3_^–^ secretion (Abdulnour-Nakhoul et al., 2011).

### Na^+^/H^+^ Exchangers

Na^+^/H^+^ exchangers (NHEs) are electroneutral exchangers with a stoichiometry of 1 Na^+^:1 H^+^. For the transport of Na^+^ and H^+^, NHEs use the energy that is provided by the electrochemical Na^+^ gradient maintained by the Na^+^/K^+^-ATPase. The N-terminal membrane domain of the exchanger is responsible for the transport of ions, whereas NHE is regulated through the C-terminal cytoplasmic domain (Slepkov et al., 2007). NHEs are involved in a wide range of biological functions, such as cell proliferation (Grinstein et al., 1989) cell migration (Denker et al., 2000) and cell volume regulation (Demaurex and Grinstein, 1994). One of their most important roles is cell alkalisation via mediating the exchange of intracellular H^+^ for extracellular Na^+^. Excess Na^+^ is removed from the cell by the Na^+^/K^+^-ATPase and Na^+^/Ca^2+^ exchanger (Slepkov et al., 2007). NHEs are encoded by the *SLC9A* gene family, which includes nine members (NHE1–NHE9). NHE1 is ubiquitously expressed, while the other isoforms are restricted to a specific tissue. Abnormal function of NHEs is associated with a number of neurological and cardiac diseases (Donowitz et al., 2013). Most NHEs are activated by acidification (Lacroix et al., 2004) and can be blocked by amiloride (Kleyman and Cragoe, 1988). Early micro-electrode studies on rabbit oesophagus have described an amiloride-sensitive short-circuit current at the serosal and mucosal sides of the oesophagus (Powell et al., 1975). Subsequently, the presence of a Na^+^/H^+^ exchange mechanism was proved by Layden et al. (1990). They identified a Na^+^-dependent, alkalizing transport system in rabbit oesophageal cells which was sensitive to amiloride and was activated at physiological pH ranges (Layden et al., 1990). Further functional studies have confirmed the presence of NHEs in rabbits (Layden et al., 1992; Abdulnour-Nakhoul et al., 1999) and humans (Tobey et al., 1998) where pH_i_ regeneration depends on extracellular Na^+^ and occurs through the basolateral membrane (Abdulnour-Nakhoul et al., 1999). Reverse transcription-polymerase chain reaction (RT-PCR) and immunoblot analysis confirm the presence of NHE1 in rat and rabbit oesophageal cells (Shallat et al., 1995). Taken together these data suggest that NHE1 is present on the basolateral membrane of rodent oesophageal cells, where it is likely to regulate the pH_i_ and protect cells against reflux-induced acidity by extruding H^+^ out of the cells. In contrast, results on NHE1 expression and function in humans are somewhat contradictory. Immunohistochemical (IHC) studies on human oesophageal tissue specimens have shown the absence or weak expression of NHE1 in normal squamous mucosa (Goldman et al., 2010; Guan et al., 2014; Laczko et al., 2016; Ariyoshi et al., 2017), while functional assays performed on the human oesophageal cell line and primary cultures detect a high degree of NHE activity (Tobey et al., 1998; Fujiwara et al., 2005). Even if NHE is present on OECs under normal conditions, the role of NHE in the oesophagus is much more important under pathological conditions as detailed below.

### Cl^–^/HCO_3_^–^ Exchangers and Na^+^/HCO_3_^–^ Co-Transporters

Cl^–^/HCO_3_^–^ exchangers or AEs mediate Cl^–^ and HCO_3_^–^ transport through the plasma membrane (Bonar and Casey, 2008). AEs either alkalise or acidify cells, depending on the direction of HCO_3_^–^ transport. The AE family comprises a large number of transporters, which are encoded by two gene families (*SLC4A* and *SLC26A*) (Bonar and Casey, 2008). The *SLC4A* family comprises three transporter types: Na^+^/HCO_3_^–^ co-transporters (NBCs), Na^+^-dependent Cl^–^/HCO_3_^–^ exchangers (NDCBE and NCBE) and Na^+^-independent Cl^–^/HCO_3_^–^ exchangers (AE1, AE2, and AE3). Five members of the *SLC26A* family (*SLC26A3*, *SLC26A4*, *SLC26A6*, *SLC26A7*, and *SLC26A9*) differ most in their different Cl^–^: HCO_3_^–^ stoichiometry and therefore in their electrogenicity. AE family members differ in many aspects, such as tissue expression, cell surface localization and physiological function. Abnormal expression of *SLC26A2-4* genes associated with rare, recessive diseases, such as diastrophic dysplasia, congenital chloride diarrhea or Pendred syndrome, respectively (Kere, 2006).

Two types of AE are found in oesophageal SECs (Tobey et al., 1993): a Na^+^-dependent AE and a Na^+^-independent Cl^–^/HCO_3_^–^ exchanger. Although both transporters are electroneutral, their effects are opposite. The Na^+^-dependent AE plays an important role in cell alkalisation, along with the NHE, while the Na^+^-independent AE acidifies cells, preventing over-alkalisation. Na^+^-independent AEs mediate the exchange of 1 Cl^–^ for 1 HCO_3_^–^, while Na^+^-dependent AEs transport Na^+^, in addition to HCO_3_^–^ and Cl^–^. Both Na^+^-dependent and Na^+^-independent AEs localize to the basolateral membrane and conduct HCO_3_^–^ transport from or toward the blood (Layden et al., 1992; Tobey et al., 1993). Oesophageal HCO_3_^–^ secretion is much more related to SMGs (Abdulnour-Nakhoul et al., 2005, 2011). A basal and carbachol-stimulated movement of HCO_3_^–^ was detected which was inhibited by DIDS in perfused pig oesophagus. IHC staining showed the presence of an NBC and AE2 on intra- and interlobular ducts and serous demilunes, indicating that HCO_3_^–^ secretion is probably due to these two transporters. These transporters are localized to the basolateral membrane of ductal cells, and they presumably provide HCO_3_^–^ and Cl^–^ for luminal HCO_3_^–^ secretion (Abdulnour-Nakhoul et al., 2011). In contrast, at the luminal membrane of interlobular ducts, studies have shown the messenger RNA (mRNA) and protein expression of Slc26A6, which mediate HCO_3_^–^ transport into the lumen, in cooperation with the CFTR Cl^–^ channel (Abdulnour-Nakhoul et al., 2011). Luminal HCO_3_^–^ secretion is believed to protect the ooesophagus by neutralizing the acidic refluxate.

### Other Ion Transporters

Awayda et al. (2004) identified non-selective cation conductance at the apical membrane of rabbit OECs which is equally permeable to monovalent cations, such as Li^+^, Na^+^, and K^+^ (Awayda et al., 2004). In addition, western blotting showed that this non-selective cation channel contains all three sub-groups of the epithelial Na channel (ENaC), in both native rabbit OE and HET-1A cells, although it does not have the characteristics of epithelial Na^+^ channels and is not inhibited by amiloride. The ENaC mediates cation uptake, although its exact function is unknown. In addition, similarly, to other epithelial cells, the Na^+^ gradient across the basolateral membrane is maintained by Na^+^/K^+^-ATPase whose presence has been detected on both squamous cells (Orlando et al., 1981, 1984) and on the basolateral membrane of acini and ductal cells of SMGs (Abdulnour-Nakhoul et al., 2011).

**FIGURE 2:**
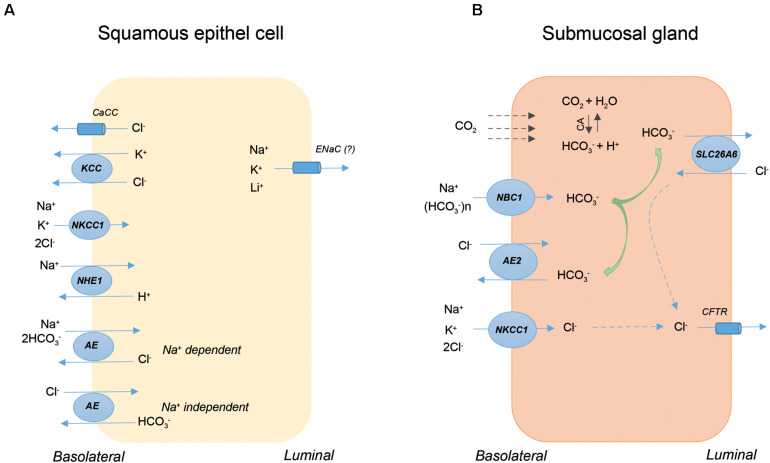
The presence and localization of ion transporters on squamous epithelial cells (SECs) and submucosal glands (SMGs). The schematic diagram shows a rabbit SEC **(A)** and a ductal cell of pig SMG **(B)**. AE, anion exchanger; CA, carbonic anhydrase; CaCC, Ca^2+^-activated Cl^–^ channel; CFTR, cystic fibrosis transmembrane conductance regulator; KCC, KCl co-transporter; NBC, Na^+^/HCO_3_^–^ co-transporter; NHE, Na^+^/H^+^ exchanger; NKCC, Na^+^/K^+^/2Cl^–^ co-transporter.

Figure 2 shows the presence and localization of ion transporters on rabbit SECs (Figure 2A) and pig SMGs (Figure 2B). In the case of SECs, transporters are mainly localized to the basolateral membrane, and their primary role is to increase the resistance of these cells to acidic reflux by restoring changes in cell volume and intracellular pH. In contrast, apical and basolateral ion transporters on SMGs are primarily involved in HCO_3_^–^ secretion, which is secreted into the lumen via the SLC26a6 AE on the apical membrane of intra-interlobular ducts. The secreted HCO_3_^–^ neutralizes the acidic gastric contents thereby largely prevents the diffusion of the proton into the oesophageal tissue. In conclusion, under physiological conditions, ion transport processes through the SECs and SMGs represent an important defense mechanism that protects the lower layers against the harmful effect of acidic GI contents. Inadequate or altered function of these transporters plays a role in the development and/or progression of oesophageal diseases, such as EoE, BO or OC.

## Role of Ion Transporters Under Pathological Conditions

### Eosinophilic Oesophagitis

Eosinophilic oesophagitis is a chronic inflammatory disease induced by food antigens (Furuta and Katzka, 2015). EoE is characterized by basal zone hyperplasia (BZH), dilated intercellular spaces (DISs) and the filtration of eosinophilic granulocytes into the oesophageal mucosa (Collins, 2008). Interleukin 13 (IL-13) plays a major role in EoE pathology. IL-13 is highly up-regulated in inflamed tissue and regulates the expression of several key molecules (Blanchard et al., 2010). Studies have reported altered expression of two ion transporters [NHE3 and anoctamin 1 (ANO1)] in EoE (Zeng et al., 2018; Vanoni et al., 2020). The mRNA and protein expression of both NHE3 and ANO1 significantly increases in biopsy samples obtained from EoE patients and in IL-13-treated OECs and model systems. Both *NHE3* and *ANO1* are up-regulated by the transcription factor signal transducer and activator of transcription 6 (STAT6) and are positively correlated with disease progression (Zeng et al., 2018; Vanoni et al., 2020). Pharmacological NHE3 inhibition blocks IL-13-induced DIS formation, indicating the involvement of NHE3 in DIS formation (Zeng et al., 2018). NHE3 mediates the exchange of intracellular H^+^ with extracellular Na^+^. Proton accumulation in the intercellular space creates osmotic conditions which favor water transport and, as a result, DIS formation (Zeng et al., 2018). In contrast to NHE3, increased ANO1 expression is associated with BZH (Vanoni et al., 2020). ANO1 is an apical Ca^2+^-activated Cl^–^ channel which is also permeable to HCO3–(Jung et al., 2013). Application of ANO1 inhibitor or short hairpin RNA (shRNA) shows that this Cl^–^ channel interacts with cell cycle regulatory proteins, such as TP63 and phosphorylated cyclin-dependent kinase 2 (p-CDK2), thereby enhancing cell proliferation. In addition, Cl^–^ efflux through ANO1 likely plays a role in intracellular Cl^–^ depletion, which is a prerequisite for cell division. These results indicate that both NHE3 and ANO1 are part of the IL-13-mediated transcriptional cascade, which leads to the histopathological features of EoE. Therefore, inhibition of these transporters could be a potential therapeutic target for EoE treatment (Vanoni et al., 2020).

### Gastro-Oesophageal Reflux Disease and Barrett’s Oesophagus

Barrett’s oesophagus is a condition in which the normal squamous epithelium is replaced by columnar cells (Spechler and Souza, 2014). It is the result of long-term gastro-oesophageal reflux disease (GERD) and a type of adaptation to the altered environment (Spechler and Souza, 2014). Many factors play a role in BO development, although the exact underlying mechanism is unclear. Several studies have been conducted in which normal epithelial cells were exposed to acidic pH, bile acids or a combination of the two and molecular changes and cell survival were investigated. Lin et al. (2008) identified DIDS- and ethyl-isopropyl amiloride (EIPA)-sensitive and HCO_3_^–^-dependent transport mechanisms in rat oesophageal tissue upon HCl administration, indicating the involvement of an NHE and one of the SLC26 AEs in the stimulatory effect of HCl (Lin et al., 2008). Increased ionic movement due to acid exposure is likely a protective mechanism and might play a role in the GERD pathomechanism. Acidic pH decreases cell viability and increases reactive oxygen species production in normal OECs, in which NHE1 plays a protective role (Park et al., 2015). Similar results have been found in human oesophageal cell lines, in which the cytoprotective effect of epidermal growth factor against acid-induced cell damage is mediated by NHE1 through the Ca^2+^/calmodulin and protein kinase C (PKC) pathway (Fujiwara et al., 2005). Increased NHE1 expression is found in oesophagitis, GERD and BO, although it is unclear whether increased NHE1 levels are the consequence or the cause of these diseases (Siddique and Khan, 2003; Fujiwara et al., 2005; Goldman et al., 2010; Laczko et al., 2016). According to Siddique and Khan (2003) NHE1 is involved in GERD development, presumably by acidifying the extracellular space (Siddique and Khan, 2003). The authors also found that in patients taking H2 receptor blockers, the mRNA level of NHE1 is significantly lower compared to untreated or antacid-treated GERD patients, assuming a specific interaction between NHE1 and the histamine receptor in GERD. In contrast, the increased NHE1 expression in BO is more likely part of a defense or adaptive mechanism which protects cells against acid reflux (Goldman et al., 2010). This hypothesis is also supported by another study in which bile treatment alone or in combination with acid increased NHE1 expression in metaplastic (CP-A) and dysplastic (CP-D) oesophageal cell lines (Laczko et al., 2016). Interestingly, NHE activity decreased in CP-A as a result of treatment but increased in CP-D cells in a dose-dependent manner. The reason probably is that CP-D cells are in a more advanced state and are more frequently exposed to acid or biliary reflux under pathological conditions. In addition to NHE1, NHE2, NBC, and Slc26a6 expression also increased in intestinal and non-intestinal metaplasia, which probably further enhances cellular resistance against reflux-induced cell damage (Laczko et al., 2016). These results suggest that increased NHE expression found in BO is essentially a defense mechanism by which metaplastic tissue tries to adapt to the altered environment. Inhibition of the transporter promotes tumorigenesis because of a decrease in intracellular pH.

Transient receptor potential channels (TRP) is a multifamily of integral membrane proteins that found on several tissues and cell types in animals (Nilius and Owsianik, 2011). TRP channels function as non-selective cation channels and are permeable to Na^+^; Ca^2+^ and Mg^2^. The TRP family can be subdivided into seven subfamilies: TRPC (canonical), TRPV (vanilloid), TRPM (melastatin), TRPP (polycystin), TRPML (mucolipin), TRPA (ankyrin), and TRPN (NOMPC-like). These channels mediate a wide range of physiological functions, such as sensations of temperature, pain, tastes, vision, olfaction, hearing and intracellular ion homeostasis. Some TRP channels act as a thermo-; osmolarity- or voltage sensor, and some of them activated by membrane stretch or cytoplasm volume changes. The activated TRP channels cause depolarisation of the cell membranes, which is essential in the development of action potential in excitable cells (Nilius and Owsianik, 2011). The role of the TRP channels in the non-excitable cells is mostly to mediate the ion homeostasis of the cells (Yu et al., 2016). TRPV1 acts as molecular acid sensor in the oesophagus which activated by acidosis (pH level under 6) (Holzer, 2008). TRPV1 is overexpressed in the oesophageal mucosa of patients with non-erosive reflux disease (NERD) and erosive esophagitis and also in animal models of NERD (Guarino et al., 2010; Silva et al., 2018). Increased levels of TRPV1 was also observed in stress-induced oesophageal inflammation (Wulamu et al., 2019). The role of TRPV1 in inflammation can be explained by the fact that activation of the channel promotes the secretion of inflammatory mediators (platelet-activating factor (PAF), Il- 1 β, Il-6) that initiates the inflammatory cascade in the mucosa of the oesophagus. Therefore, inhibition of TRPV1 could be beneficial in inflammatory conditions. Recently, Zhang et al. have shown that menthol inhibits acid-induced inflammation by inhibiting TRPV1 expression and TRPV1-mediated Ca^2+^ influx in oesophageal epithelial cells (Zhang et al., 2020). TRPV4 is also a non-selective cation channel activated by mechanical, thermal, and chemical stimuli. Acid-sensitive TRPV4 channels are mainly expressed in the epithelial cells of the basal and intermediate layers of the oesophagus (Shikano et al., 2011). Ueda et al. showed the presence of TRPV4 in human oesophageal biopsy samples and HET-1A cell line. They demonstrated that activation of TRPV4 increases intracellular Ca^2+^ level and decreases Il-8 production that plays a role in different cellular functions, such as cell division or viability (Ueda et al., 2011). The presence of functionally active TRPV4 has also been shown in mouse oesophageal keratinocytes, where activation of the channel caused ATP release which affects the sensory nerves and might also be related to the pathophysiology of NERD (Mihara et al., 2011; Suzuki et al., 2015).

### Oesophageal Cancer

Oesophageal cancer is a malignant neoplasm, and the sixth-leading cause of cancer-related death worldwide (Klingelhofer et al., 2019). The two most common types of OC are oesophageal squamous cell carcinoma (ESCC) and oesophageal adenocarcinoma (EAC) (Enzinger and Mayer, 2003). Current treatment of OC includes surgery, chemo- or radiotherapy or the combination of the three. Although OC treatment and diagnosis have advanced, the 5-year survival rate is between 15 and 20% and recurrence is frequent (Shapiro et al., 2015). Therefore, there is an urgent need to develop new therapies that can complement existing treatments or allow individual therapies. Disturbances of ion transport processes are considered to be of great importance in cancer research (Pedersen and Stock, 2013; Djamgoz et al., 2014; Oosterwijk and Gillies, 2014; Stock and Schwab, 2015; Martial, 2016). Ion transporters are not only involved in tumorigenesis by altering the membrane permeability of certain ions, but some transporters interact with other signaling molecules (kinases, transcription factors) that influence the cell cycle or the metastatic potential of the cells. In recent years, several transporters and ion channels have been identified whose expression is altered in OC. In the following, those ion transporters are discussed, that might provide a target point for novel treatment or serve as prognostic markers for ESCC or EAC (Supplementary Table S1).

### K^+^ Channels

Ding et al. (2008a, b) identified two voltage-gated K^+^ channels, Eag1 (KV10.1, KCNH1) and hERG1 (KV11.1, KCNH2), which are aberrantly expressed in ESCC and are correlated with poor prognosis (Ding et al., 2008a, b). *Human ether a-go-go-related gene 1* (*hERG1*) over-expression has been found in BO and even more in dysplasia and adenocarcinoma, indicating that *hERG1* expression increases during BO progression to EAC (Lastraioli et al., 2006). To support this observation, Lastraioli et al. (2016) conducted a comprehensive study on *hERG1* expression in both mice and humans. The authors found increased *hERG1* expression in two different BO mice models. They also found that surgically induced BO more frequently develops in mice with *hERG1* over-expression. The significance of hERG1 in BO progression to EAC has also been demonstrated in human biopsy samples obtained from BO patients. Using monoclonal antibody against hERG1, the 10-year follow-up showed that BO patients with increased hERG1 expression have a higher risk of EAC development, indicating that hERG1 can be used as a biomarker to predict disease progression (Lastraioli et al., 2016).

#### Inhibitors and Therapeutic Perspectives

Potassium channel blockers are most commonly used as antiarrhythmic agents, however, research in recent years has shown that there is a growing need for K^+^ channel inhibitors in cancer therapy as well. Currently, AZD9291 is used in the treatment of non-small cell lung cancer (Janne et al., 2015). AZD9291 is an epidermal growth factor receptor (EGFR) tyrosine kinase inhibitor (TKI) that is able to inhibit hERG channels. Another EGFR TKI, gefitinib has also been proved to be effective in non-small cell lung cancer patients and has shown to be beneficial in breast cancer as well (Garcia-Quiroz et al., 2019). Experimental data demonstrated that gefitinib dose-dependently inhibited the proliferation of cancer cells and combined administration of this drug with the antihistamine, astemizole resulted in a synergistic effect (Garcia-Quiroz et al., 2019). Although the two EGFR TKIs inhibit different channels, AZD9291 is specific for hERG channel, while gefitinib for Eag1, they have similar mechanisms of action, both of them prevent the channel phosphorylation which is required for the activation of the channels (Zhang et al., 2008; Wu et al., 2012).

### ANO1

Shang et al. (2016) reported a correlation between anoctamin-1 (ANO1) expression and primary oesophageal tumor in two independent cohorts. The authors detected strong ANO1 staining in the tumor region of surgical specimens obtained from ESCC patients, while the adjacent normal tissue and endoscopic biopsies from normal mucosa and chronic oesophagitis were completely negative for this Cl^–^ channel (Shang et al., 2016). In addition, ANO1 expression is correlated with the malignant progression of precancerous oesophageal lesions, indicating that ANO1 can be used as a biomarker to predict disease progression. Shi et al. (2009) identified genes whose expression is altered in ESCC and dysplasia. They found increased ANO1 levels at both mRNA and protein levels in ESCC and dysplastic samples. In addition, they found a correlation between ANO1 expression and tumor progression (Shi et al., 2004). Interestingly, in the mRNA expression of ANO1, the authors found no significant difference between normal mucosa and oesophagitis (Shi et al., 2009) although Vanoni et al. (2020) have shown increased ANO1 expression in EoE. Genetic inhibition of ANO1 decreases proliferation in ESCC cell lines, confirming the role of ANO1 in cell division. The mechanism underlying ANO1-induced promotion of cell proliferation is unclear, although, in head and neck squamous cell carcinoma, activation of the mitogen-activated protein kinase (MAPK)/extracellular signal-regulated kinase (ERK) signaling pathway is involved in the proliferative effect of ANO1 (Duvvuri et al., 2012).

#### Inhibitors and Therapeutic Perspectives

Several inhibitors are known that can inhibit ANO1 with greater or lesser potency or selectivity, such as the small molecule inhibitor, Ani9 (Seo et al., 2016) the benzofuran derivative, benzbromarone (Huang et al., 2012), the flavonoid, luteolin (Seo et al., 2017) the aminophenylthiazole, [T16A(inh)-A01] or the arylaminothiophene, CaCC(inh)-A01 (Namkung et al., 2011). Among them, luteolin has been shown as potential anticancer therapeutic agents for the treatment of prostate cancer (Seo et al., 2017). This inhibitor not only blocks ANO1 activity but also decreases its protein expression. Using PC-3, prostate cancer cell line, administration of luteolin reduced the proliferation and migration of the cells (Seo et al., 2017). Similar results were observed with Ani9, T16A(inh)-A01 and CaCC(inh)-A01 inhibitors, which also reduced PC-3 cell proliferation and induced apoptosis, by a TNF-α-mediated signaling pathway (Song et al., 2018). ANO1 inhibitors have also been shown to be beneficial in ovarian cancer, where administration of LY294002 reduced the growth of cancer cells by inhibiting the PI3K/Akt signaling pathway (Liu et al., 2019). The mechanism of action of ANO1 inhibitors is not yet known, and there is no evidence to date that it has been used in the treatment of inflammatory or cancerous diseases of the oesophagus, but it could be a promising target.

### AEs

Shiozaki et al. (2017) characterized the role of AE1 and AE2 in ESCC tumorigenesis. Analysis of ESCC samples showed that the AE1 staining intensity depends on the tumor length, while the focal or diffuse distribution of AE1 is correlated with the histological type and pT stage (Shiozaki et al., 2017). With regard to 5-year survival, the staining score was not correlated with prognosis, although diffuse AE1 expression was associated with poor survival in advanced stages (Shiozaki et al., 2017). AE1 expression has been found in ESCC cell lines (TE5, TE8, TE9, and KYSE150), where it is involved in cell cycle regulation and the proliferation, apoptosis, migration and invasion of ESCC cells via the MAPK and Hedgehog signaling pathways (Shiozaki et al., 2017). In contrast to AE1, decreased AE2 expression is associated with poor prognosis in ESCC patients (Shiozaki et al., 2018). There is a correlation between the AE2 staining score and the pT stage. With regard to survival, low AE2 expression predicts a worse prognosis. Interestingly, these correlations were observed only in the invasive front of ESCC, not in the entire tumor. In contrast to AE1, AE2 expression was found in KYSE170, and TE13 ESCC cell lines and AE2 knock-down decreased apoptosis and increased migration and invasion of ESCC cells, indicating that AE2 regulates cell survival and cellular movements. Studies have also demonstrated that the effect of AE2 is mediated by matrix metalloproteinases (MMPs) and their inhibitors. Altered AE2 expression is believed to affect tumorigenesis by altering intracellular pH. Decreased AE2 expression favors intracellular alkalisation, which promotes cancer cell metabolism (Persi et al., 2018).

#### Inhibitors and Therapeutic Perspectives

The most commonly used inhibitors of anion transporters are the stilbene derivatives, DIDS, its reduced form dihydroDIDS (H2DIDS) and 4-acetamido-4′-isothiocyanatostilbene-2,2′-disulfonic acid (SITS). The negatively charged sulfonate group of these inhibitors interact electrostatically with the anion binding site of the transporter, resulting in a rapid, reversible inhibition, followed by a slower covalent interaction between the isothiocyanate group of the inhibitor and the free amino acids of the AE. These inhibitors have low specificity and inhibit individual isoforms with different potency (Humphreys et al., 1994; Alper et al., 1998). There are also differences in the efficacy of inhibitors between species and cell types. The oxonol dye, diBA(5)C4 has been shown to inhibit the AE1 isoform more effectively than AE2, while polyaminosterols have higher selectivity to AE2 (Alper et al., 1998). Among the more recently discovered AE inhibitors, 4,8-dimethyl-7-(*m*-bromobenzyloxy)-coumarin-3-acetic acid has been shown to be able to completely and selectively inhibit the SLC26A3 exchanger, and the use of this agent showed a beneficial effect on constipation in a mouse model (Lee et al., 2019) although, to our knowledge, there is currently no AE inhibitor which enters or underwent in clinical trials.

### KCC3 and NKCC1

Among KCC isoforms, KCC3 expression is associated with OC tumorigenesis (Shiozaki et al., 2014b). The presence of KCC3 in the invasive front of ESCC is associated with a low survival rate post-operatively. The mechanism underlying KCC3’s effect on tumor progression is unclear, but studies using ESCC cell lines have shown that KCC3 is essential for the invasiveness of cancer cells (Shiozaki et al., 2014b). NKCC1 is also a biomarker in ESCC. NKCC1 expression is closely related to the differentiation of ESCC cells, and genetic or pharmacologic inhibition of NKCC1 in ESCC cell lines significantly decreases the rate of cell proliferation, indicating that NKCC1 is involved in cell cycle regulation (Shiozaki et al., 2014a).

#### Inhibitors and Therapeutic Perspectives

The loop diuretics, bumetanide and furosemide, are the most commonly used inhibitors of NKCCs. Both inhibitors are able to inhibit the co-transporter even at very low concentrations (0.5–5 μM). Diuretics have long been used to treat fluid retention, and in recent years their use has also emerged in the treatment of certain neurological disorders. Therefore, several clinical studies have been conducted to test the effect of bumetanide in schizophrenia, epilepsy or Parkinson’s disease (Ben-Ari, 2017). Although, the clinical use of this inhibitor is limited due to its ionized state at physiological pH and its high binding to plasma proteins. Recent studies suggest that azosemide is more potent than bumetanide and has better pharmacokinetic properties (Hampel et al., 2018). Bumetanide and furosemide at higher concentrations (100 μM–1 mM) inhibit KCCs as well. In addition, KCC also sensitive to DIDS, H2DIDS, and SITS, although the inhibitory effect of these stilbene derivatives highly depends on external K^+^ (Delpire and Lauf, 1992). Currently, the most commonly used KCC inhibitor is dihydroindenyloxy alkanoic acid (DIOA), which selectively inhibits the transporter. The large-scale high-throughput screen on HEK293 cells identified more potent inhibitors, such as ML077 or VU0463271, (Delpire and Weaver, 2016) although none of these compounds is currently under clinical development.

### NHE

Among ion transporters, the expression of NHEs, especially NHE1, alters in a metaplasia-dysplasia-adenocarcinoma sequence. Recent studies have demonstrated that NHE1 also plays a significant role in BO progression to EAC. Fitzgerald et al. (1998) showed that NHE1 is involved in acid-induced cell proliferation in BO biopsy samples in a PKC-dependent manner (Fitzgerald et al., 1998). The cell proliferation-inducing effect of NHE1 is explained by increased NHE1 activity at acidic pH, which likely affects cell division by altering intracellular pH. In contrast, bile acids alone or in combination with acid decrease NHE1 activity in nitric oxide– and nitric oxide synthase–dependent manner in BO-derived CP-A cells, leading to cellular acidosis (Goldman et al., 2010). The decrease in intracellular pH favors DNA damage, which increases the incidence of mutations and, therefore, the risk of EAC progression (Goldman et al., 2010). However, this workgroup also showed that bile acid damages lysosomes as well in JH-EsoAd1 and CP-A cells and the acidic content released from them increases NHE activity, which forms an ionic environment that favors to apoptotic processes. Based on these findings, it is hypothesized that by the inhibition of NHE, the cells avoid apoptosis, increasing the risk of cancer transformation (Goldman et al., 2011). The role of NHE1 in OC is contradictory. Guan et al. (2014) have shown that NHE1 is over-expressed in EAC and inhibition of NHE1 expression or blocking of NHE activity by amiloride decreases cancer cell proliferation and induces apoptosis in both EAC and ESCC cell lines and in nude mice xenografts. Moreover, amiloride in combination with guggulsterone (antagonist of frasenoid X receptor) proved to be even more effective than amiloride alone. These results indicate that contrast to metaplastic cells, inhibition of NHE does not enhance tumor progression but, on the contrary, inhibits it, suggesting that NHE behaves differently in cancerous and metaplastic cells. In contrast, Ariyoshi et al. (2017) found that NHE1 inhibition favors the proliferation, migration and invasion of ESCC cells because of the up-regulation of epithelial-mesenchymal transformation (EMT) markers and the inhibition of Notch signaling. The 5-year overall survival (OS) was significantly higher in ESCC patients with high NHE1 expression, indicating the possibility of using NHE1 as a prognostic marker (Ariyoshi et al., 2017).

#### Inhibitors and Therapeutic Perspectives

The most commonly used NHE1 inhibitors are amiloride and its derivatives such as EIPA (5-(N-ethyl-N-isopropyl) amiloride), DMA (5-(N,N-dimethyl) amiloride) and HMA (5-(N,N-hexamethylene) amiloride) that belongs to the pyrazinoylguanidine-type inhibitors, as well as the more specific cariporide and epripride that belongs to the benzoylguanidine-type inhibitors (Rolver et al., 2020). In the case of both types of inhibitors, guanidine binds to the sodium binding site of the exchanger, thereby competes with the sodium for the binding site (Frelin et al., 1986). Numerous studies have suggested the use of NHE inhibitors in the treatment of various types of cancer, such as breast or colon cancer (Miraglia et al., 2005; Rolver et al., 2020), however, clinical evaluation of NHE inhibitors is currently limited to cardiac disease, particularly in connection with ischemia-reperfusion injury, in which the cardioprotective effect of cariporide and eniporide was demonstrated in two clinical trials (Chaitman, 2003; Mentzer et al., 2008).

### Ca^2+^ Channels

Orai-1 is the pore-forming subunit of the calcium-release activated channel. By interacting with the Ca^2+^ sensor stromal interaction molecule 1 (STIM-1), Orai-1 mediates store-operated Ca^2+^ entry (SOCE). Orai-1 is involved in tumorigenesis in many cancer types, including OC (Vashisht et al., 2015). Zhu et al. (2014) reported increased Orai-1 expression in tumor tissues obtained from ESCC patients. The Orai-1 expression is correlated with clinicopathological features, such as histological grade, T-stage classification and lymph node metastasis, and poor OS and recurrence-free survival rates. Increased Orai-1 expression and intense oscillatory Ca^2+^ activities are also found in ESCC cell lines, which probably plays a role in carcinogenesis. The role of Orai-1 in tumorigenesis has been confirmed using cell-based assays in which Orai-1 knock-down decreased the proliferation, migration and invasion of ESCC cells and tumor growth in nude mice xenografts (Zhu et al., 2014). The proliferative effect of Orai-1 might be inhibited by zinc, which selectively interacts with the histidine residue of Orai-1 and inhibits SOCE and Ca^2+^ oscillations in ESCC cells (Choi et al., 2018). RP4010, a specific Orai-1 inhibitor, also inhibits tumor growth via an unknown mechanism and is a potential therapeutic or adjuvant drug for ESCC treatment (Cui et al., 2018). The role of voltage-gated Ca^2+^ channels has also been raised in relation to OC. Voltage-gated calcium channels are comprised of heteromultimeric complexes that involve α1 protein in combination with various auxiliary subunits, β, α2δ, and γ protein (Walker and De Waard, 1998). Four α2δ subunit genes have been identified (*CACNA2D1-4)* (Qin et al., 2002). The α2δ-3 subunit (CACNA2D3) is responsible for Ca^2+^ influx, and its tumor suppressor function was also characterized. It has been shown, that *CACNA2D3* induces apoptosis, reduces cell motility, and provokes cell cycle arrest in G1 phase through transactivating p53/p21 signaling pathways (Lipskaia and Lompre, 2004;Li et al., 2013). The potential tumor suppressor role of *CACNA2D3* in the pathomechanism of malignancies has been mentioned in several studies. Considering the GI tract, the promoter methylation of this gene is in association with poor prognosis of gastric cancer (Wanajo et al., 2008). Li et al. showed that downregulation of *CACNA2D3* could be detected in ESCC patients that showed a positive correlation with a poor progression and low overall survival rate (Li et al., 2013). A recent study indicates, that overexpression of CACNA2D3 can restore chemosensitivity of ESCC cells to cisplatin via inhibition of PI3K/Akt pathway, which is a promising finding regarding the therapy of ESCC (Nie et al., 2019).

TRPC6 proteins are non-selective cation channels, which are permeable to cations (Ca^2+^, Na ^+^, K^+^, Cs^+^ and Ba^2+^) iron, zinc and N-methyl-D-glucamine (Bouron et al., 2016). TRPC6 seems to be ubiquitous in the human body and involved in the regulation of intracellular Ca^2+^ concentration (Hofmann et al., 2000). There are several activators of the TRPC6 channels, such as extracellular Ca^2+^ concentration,(Shi et al., 2004) diacylglycerol (DAG) and its analogs, such as 1-oleoyl-2-acetyl-sn-glycerol, and arachidonic acids (Aires et al., 2007) and hyperforin (Leuner et al., 2007). Literature data indicate that TRPC6 is associated with tumorigenesis, tumor growth and elevated TRPC6 expression was detected in many cancer types (Santoni and Farfariello, 2011). It has been reported that TRPC6 expression significantly higher in ESCC patients, and it correlates with poor progression (Shi et al., 2009; Zhang et al., 2013). Shi et al. have shown, that inhibition of TRPC6 resulted in cell cycle arrest at G2 phase, reduced cell growth in ESCC cells and the formation of tumor in nude mice (Shi et al., 2009). Evaluating this finding (Ding et al., 2010) have raised the possibility that inhibition of TRPC6 can increase the radiosensitivity of tumor cells and also inhibits angiogenesis in the therapy of ESCC. Similar results have been found by Zhang et al. Using an ESCC cell line, Eca109 they showed that administration of the TRPC inhibitor, SKF96365 arrested the cell cycle at G2/M phase, and in combination with radiation it significantly reduced *in vitro* cell proliferation and the tumor size in nude mice (Zhang et al., 2017).

The role of TRPVs has arisen primarily in the inflammatory diseases of the oesophagus (GERD, NERD); although Huang et al. have also shown its importance in ESCC (Huang et al., 2019). They found that both TRPV1 and -4 are up-regulated in ESCC, and overactivation of these channels can modulate the proliferation and migration of ESCC cells, by the regulation of intracellular Ca^2+^ concentration (Huang et al., 2019).

#### Inhibitors and Therapeutic Perspectives

Ca^2+^ channels are aberrantly regulated in most of the cancer cells; therefore, great efforts are made to develop novel drugs that are able to modulate the function of these channels. Inhibition of Ca^2+^ channels is a promising target in cancer therapy, and there are Ca^2+^ channel blockers that have already undergone clinical trials or are under clinical development. The specific Orai-1 inhibitor, RP4010 completed a Phase I/Ib clinical trial in which the safety and efficacy of RP4010 were evaluated in lymphoma. In addition, RP4010 significantly reduced cell division, especially in combination with anticancer agents in pancreatic ductal adenocarcinoma (Khan et al., 2020). The recently identified SOR-C13 is an inhibitor of TRPV6 channels that selectively binds to the channel and prevents the influx of Ca^2+^ into the cells. SOR-C13 has been shown to be effective in ovarian cancer (Xue et al., 2018) and is currently undergoing Phase I clinical trials to determine the best dose and side effect profile of this drug, in advanced refractory solid tumors.

### Aquaporins

Ion movement is accompanied by water movement. Transcellular water transport is mediated by aquaporins (AQPs). The main driving force of AQPs is the osmotic difference between the intra- and the extracellular space. In addition to water transport, AQPs are involved in the transport of smaller molecules or function as ion channels. Several mechanisms have been described through which AQPs enhance tumor cell migration and invasion (De Ieso and Yool, 2018). AQP1 facilitates hypoxia-induced angiogenesis, AQP1, -3, -4, -5, and -9 are involved in epithelial-mesenchymal transition and extracellular matrix degradation, whereas AQP1-4 enhance cell adhesion (De Ieso and Yool, 2018). Of the 13 isoforms currently identified, AQP1, -3, -5, and -8 are associated with OC. AQP1 is widely expressed in many tissues and is a potential biomarker in breast cancer (Qin et al., 2016). Yamazato et al. (2018) found a correlation between AQP1 expression and the outcome of ESCC patients (Yamazato et al., 2018). The authors showed that in ESCC patients with high AQP1 expression in the cytoplasm, the 5-year survival rate is lower compared to ESCC patients in whom AQP1 is localized to the nuclear membrane. AQP1 knock-down in ESCC cell lines inhibits cell proliferation and migration and induces apoptosis. In addition, microarray analysis shows an interaction between AQP1 and death-receptor signaling pathway-related genes, supporting the importance of AQP1 in tumorigenesis. Similar results were observed for AQP3 (Kusayama et al., 2011; Niu et al., 2012). AQP3 is highly expressed in ESCC, and the absence or decreased function of AQP3 decreases adhesion of cancerous cells via inhibition of integrins and the focal adhesion kinase–mitogen-activated protein kinase (FAK-MAPK) signaling pathway. Inhibition of cell adhesion is associated with decreased cell growth and apoptosis, indicating the critical role of AQP3 in cell survival. Increased expression of AQP3 in ESCC and EAC has also been demonstrated by Niu et al. (2012). Studies have also reported the importance of AQP5 in ESCC (Shimizu et al., 2014). AQP5 down-regulation inhibits cell cycle progression in ESCC cell lines, and microarray analysis shows that a lack of AQP5 affects the expression of genes involved in cellular growth. Studies have shown a correlation between the size and histological type of ESCC and AQP5 expression in human tumor tissues. In addition, the 3-year progression-free survival is lower in ESCC patients with AQP5 positivity, which raises the possibility of using AQP5 as a biomarker. AQP8 has also been implicated in the progression of OC (Chang et al., 2014). Using human ESCC cell line, Eca-109, it has been shown that the epidermal growth factor (EGF)-induced cell migration is associated with increased expression of AQP8 through the EGF receptor-ERK1/2 signal transduction pathway.

#### Inhibitors and Therapeutic Perspectives

Several inhibitors have been identified that are more or less effective in inhibiting AQPs, such as the small molecule inhibitors, TEA and acetazolamide, which can selectively and reversibly inhibit AQP1 and -4 isoforms (Abir-Awan et al., 2019). The recently discovered molecules, DFP00173 and Z433927330 that have been shown to block glyceroporins by reducing channel glycerol permeability (Abir-Awan et al., 2019). Among the heavy metal ions, Cu ions, NiCl_2_ and gold compounds proved to be effective in inhibiting AQP3 (Aikman et al., 2018). HgCl_2_ is also widely used to inhibit AQPs, however, the disadvantage of this molecule is that it blocks all AQP isoforms and mercury is highly toxic to cells due to its many side effects (Aikman et al., 2018). Furthermore, monoclonal antibodies such as anti-AQP4 have been shown to be promising agents in the treatment of neuromyelitis optica (NMO) (Tradtrantip et al., 2012) and clinical trials are in progress to evaluate the efficacy of anti-AQP4 IgG in NMO and multiple sclerosis. To the best of our knowledge, no controlled clinical trial has been conducted to date in which the effects of AQP inhibitors have been tested in oesophageal diseases.

## Conclusion

This review summarized the expression and role of ion transporters in OECs (Tables 1, 2) and described their pathological function (Supplementary Table S1). Ion transport fundamentally determines both the extra- and the intracellular pH, which affects several intracellular mechanisms, including the release of inflammatory mediators or the expression of cell cycle regulatory proteins. Research increasingly highlights the significance of ion transporters in various inflammatory and cancerous processes, and many of them have emerged as promising therapeutic targets or independent prognostic factors in cancer. This review described numerous ion transporters whose inhibition reduces proliferation and induces apoptosis of cancerous oesophageal cells (Supplementary Table S1). These findings raise the possibility that pharmacological inhibition of these ion transporters or a combination of ion transporter inhibitors with chemotherapeutic agents might provide alternative treatment options for OC patients who do not respond to standard treatment.

## Author Contributions

VV, EB, and MK contributed to conception and design of the study. EB and PH wrote separate sections of the manuscript. All authors read and approved the submitted version.

## Conflict of Interest

The authors declare that the research was conducted in the absence of any commercial or financial relationships that could be construed as a potential conflict of interest.
