# Supplementary material for: OEsophageal Ion Transport Mechanisms and Significance Under Pathological Conditions
Source: Front Physiol. 2020 Jul 16;11:855. doi: 10.3389/fphys.2020.00855 (PMC7379034; doi:10.3389/fphys.2020.00855)
Supplement: Supplementary file 1 [file Table_1.docx]

**Supplementary Table 1. Therapeutic targets and prognostic factors in oesophageal cancer (OC)**. ESCC, oesophageal squamous cell carcinoma; EAC, oesophageal adenocarcinoma; AE, anion exchanger; ANO1, anoctamine-1; AQP, aquaporin; CACNA2D3, voltage-gated calcium channel α2δ subunit 3; EGF, epidermal growth factor; EGFR, EGF receptor; EMT, epithelial–mesenchymal transformation; ERK-extracellular signal-regulated kinase; FAK, focal adhesion kinase; KCC, KCl co-transporter; MAPK, mitogen-activated protein kinase; MMPs: matrix metalloproteinases; NKCC, Na^+^/K^+^/2Cl^–^ co-transporter; NHE, Na^+^/H^+^ exchanger; PI3, phosphatidylinositol 3-kinase; TRPC, canonical transient receptor potential channels; TRPV, transient receptor potential vanilloid channels; N/A, not available.

| **Therapeutic targets and prognostic factors in oesophageal cancer (OC)** | | | | | |
| --- | --- | --- | --- | --- | --- |
| **Family** | **Member** | **Histological type of OC** | **Expression changes in cancer** | **Mechanism of action** | **Reference** |
| K^+^ channels | Eag1 | ESCC | upregulated | N/A | Ding et al. 2008a |
|  | hERG1 | ESCC | upregulated | N/A | Ding et al. 2008b |
|  |  | EAC | upregulated | N/A | Lastraioli et al., 2006 and 2016 |
| Cl^-^ channels | ANO1 | ESCC | upregulated | Activation of the MAPK/ERK signalling pathway | Shang et al., 2016; Shi et al., 2013 |
| AE | AE1 | ESCC | upregulated | Activation of the MAPK and Hedgehog signalling pathways | Shiozaki et al., 2017 |
|  | AE2 | ESCC | down-regulated | Altered expression of genes encoding MMPs and their inhibitors | Shiozaki et al., 2018 |
| KCC | KCC3 | ESCC | upregulated | N/A | Shiozaki et al., 2014b |
| NKCC | NKCC1 | ESCC | upregulated | Altered expression of G2/M checkpoint related genes | Shiozaki et al., 2014a |
| NHE | NHE1 | ESCC | upregulated | Downregulation of NHE1 alters the expression of EMT and Notch signalling-related genes and activates PI3K-AKT signal pathways | Ariyoshi et al., 2017 |
|  |  | EAC | upregulated | N/A | Guan et al., 2014 |
| Ca^2+^ channels | Orai-1 | ESCC | upregulated | Induction of hyperactive intracellular Ca^2+^ oscillations | Vashisht et al., 2015; Zhu et al., 2014; Choi et al., 2018; Cui et al., 2018 |
|  | TRPV1 | ESCC | upregulated | Elevation of [Ca^2+^]_i_ | Huang et al., 2019 |
|  | TRPV4 | ESCC | upregulated |  |  |
|  | TRPC6 | ESCC | upregulated | Elevation of [Ca^2+^]_i_ and activation of Cdc2 kinase | Shi et al. 2009 |
|  | CACNA2D3 | ESCC | downregulated | Tumorsupressor gene, transactivation of the p53/p21 signalling pathways causes apoptosis and cell cycle arrest in G1 phase | Li et al 2013, Lipskaia et al 2004 |
| AQPs | AQP1 | ESCC | upregulated | Interaction with the death-receptor signalling pathway–related genes | Yamazato et al., 2018 |
|  | AQP3 | ESCC | upregulated | Activation of the FAK-MAPK signalling pathways | Kusayama et al., 2011; Niu et al., 2011 |
|  |  | EAC | upregulated | N/A | Niu et al., 2011 |
|  | AQP5 | ESCC | upregulated | Altered expression of genes related to tumour growth and apoptosis | Shimizu et al., 2014 |
|  | AQP8 | ESCC | upregulated | AQP8 expression induced by EGF via EGFR/Erk1/2 signal transduction pathway | Chang et al., 2014 |
